# A novel role for the extracellular matrix glycoprotein‐Tenascin‐X in gastric function

**DOI:** 10.1113/JP277195

**Published:** 2019-01-23

**Authors:** Rubina Aktar, Madusha Peiris, Asma Fikree, Simon Eaton, Stamatiki Kritas, Stephen J. Kentish, Eduardo J. A. Araujo, Cristiano Bacarin, Amanda J. Page, Nicol C. Voermans, Qasim Aziz, L. Ashley Blackshaw

**Affiliations:** ^1^ Blizard Institute Queen Mary University of London London UK; ^2^ Institute of Child Health University College London London UK; ^3^ South Australian Health and Medical Research Institute University of Adelaide Australia; ^4^ Department of Histology Centre for Biological Sciences State University of Londrina Brazil; ^5^ Department of Neurology Radboud University Medical Centre Nijmegen Netherlands

**Keywords:** Gastric emptying, gastric hypersensitivity, enteric neurons

## Abstract

**Key points:**

- Tenascin X (TNX) functions in the extracellular matrix of skin and joints where it maintains correct intercellular connections and tissue architecture
- TNX is associated exclusively with vagal‐afferent endings and some myenteric neurones in mouse and human stomach, respectively.
- TNX‐deficient mice have accelerated gastric emptying and hypersensitivity of gastric vagal mechanoreceptors that can be normalized by an inhibitor of vagal‐afferent sensitivity.
- Cultured nodose ganglion neurones showed no changes in response to capsaicin, cholecystokinin and potassium chloride in TNX‐deficient mice.
- TNX‐deficient patients have upper gastric dysfunction consistent with those in a mouse model. Our translational studies suggest that abnormal gastric sensory function may explain the upper gut symptoms present in TNX deficient patients, thus making it important to study gastric physiology.
- TNX deficiency should be evaluated routinely in patients with connective tissue abnormalities, which will enable a better understanding of its role and allow targeted treatment. For example, inhibitors of vagal afferents‐baclofen could be beneficial in patients. These hypotheses need confirmation via targeted clinical trials.

**Abstract:**

Tenascin‐X (TNX) is a glycoprotein that regulates tissue structure via anti‐adhesive interactions with collagen in the extracellular matrix. TNX deficiency causes a phenotype similar to hypermobility Ehlers–Danlos syndrome involving joint hypermobility, skin hyperelasticity, pain and gastrointestinal dysfunction. Previously, we have shown that TNX is required for neural control of the bowel by a specific subtype of mainly cholinergic enteric neurones and regulates sprouting and sensitivity of nociceptive sensory endings in mouse colon. These findings correlate with symptoms shown by TNX‐deficient patients and mice. We aimed to identify whether TNX is similarly present in neural structures found in mouse and human gastric tissue. We then determined whether TNX has a functional role, specifically in gastric motor and sensory function and nodose ganglia neurones. We report that TNX was present in calretinin‐immunoreactive extrinsic nerve endings in mouse and human stomach. TNX deficient mice had accelerated gastric emptying and markedly increased vagal afferent responses to gastric distension that could be rescued with GABA_B_ receptor agonist. There were no changes in nodose ganglia excitability in TNX deficient mice, suggesting that vagal afferent responses are probably the result of altered peripheral mechanosensitivity. In TNXB‐deficient patients, significantly greater symptoms of reflux, indigestion and abdominal pain were reported. In the present study, we report the first role for TNX in gastric function. Further studies are required in TNX deficient patients to determine whether symptoms can be relieved using GABA_B_ agonists.

## Key points

- Tenascin X (TNX) functions in the extracellular matrix of skin and joints where it maintains correct intercellular connections and tissue architecture
- TNX is associated exclusively with vagal‐afferent endings and some myenteric neurones in mouse and human stomach, respectively.
- TNX‐deficient mice have accelerated gastric emptying and hypersensitivity of gastric vagal mechanoreceptors that can be normalized by an inhibitor of vagal‐afferent sensitivity.
- Cultured nodose ganglion neurones showed no changes in response to capsaicin, cholecystokinin and potassium chloride in TNX‐deficient mice.
- TNX‐deficient patients have upper gastric dysfunction consistent with those in a mouse model. Our translational studies suggest that abnormal gastric sensory function may explain the upper gut symptoms present in TNX deficient patients, thus making it important to study gastric physiology.
- TNX deficiency should be evaluated routinely in patients with connective tissue abnormalities, which will enable a better understanding of its role and allow targeted treatment. For example, inhibitors of vagal afferents‐baclofen could be beneficial in patients. These hypotheses need confirmation via targeted clinical trials.

## Introduction

Tenascin‐X (TNX) is an extracellular matrix glycoprotein belonging to a family of tenascins and other molecules that governs tissue structure (Valcourt *et al*. 2015). Its absence leads to heritable connective tissue disorders with a phenotype similar to hypermobility Ehlers–Danlos Syndrome (hEDS) (Zweers *et al*. 2004), although TNX deficient patients are now classified as a separate subgroup (Malfait *et al*. 2017). We recently showed that TNX is expressed not only in connective tissue, but also in colonic intrinsic neurones, and that it is required for normal neural control of colonic motility (Aktar *et al*. 2018). Our data suggested a direct role in cholinergic control, although we also found that absence of TNX leads to hypertrophy and hypersensitivity of nociceptive sensory nerve endings in the colon that do not themselves express TNX, therefore suggesting an indirect role in pain. These observations in knockout (KO) mice correlated closely with gastrointestinal (GI) symptoms in genetically confirmed TNX‐deficient patients with hEDS, including disrupted motility and bowel habit, as well as visceral pain (Aktar *et al*. 2018). There remains, however, a large gap in our understanding because patients with hEDS suffer from symptoms that are localized in both upper and lower GI tract. The most common upper GI symptoms include bloating, reflux, disordered gastric emptying of a meal, nausea and indigestion (Castori *et al*. 2010; Mathias *et al*. 2012; Fikree *et al*. 2014). We aimed to investigate first whether there was a systematic link between TNX gene deficiency and symptoms in humans and, second, what is the underlying mechanism. In particular, vagal afferent fibres innervating the upper GO tract play a critical role both in initiation of symptoms and reflexes controlling several functions (Page & Blackshaw, 2009), Vagal afferents are comprised of two main types: (i) mucosal endings that respond to touch and to chemical stimuli and (ii) muscular endings that respond optimally to mechanical stretch or tension (Page *et al*. 2002) These gastric mechanoreceptors have been anatomically identified as intraganglionic laminar endings (IGLE). Another population of intramuscular arrays (IMA) may also play a role in mechanosensation (Zagorodnyuk *et al*. 2001). They project to the brain stem where they provide input to central sensory pathways and motor programmes (Browning & Travagli, 2010).

We first investigated the correlation between TNX gene deficiency and symptom severity in a hEDS patient cohort. We then determined TNX expression in calretinin‐immunoreactive structures analogous with vagal afferent endings in both humans and mice. Electrophysiological recordings were used to demonstrate whether TNX KO mice have altered sensitivity of vagal afferents to gastric distension. The association of changes in vagal afferent function with overall gastrointestinal function was explored by measuring gastric emptying *in vivo*. We also investigated a means of normalizing afferent function by activation of inhibitory GABA_B_ receptors on gastric vagal afferents. What emerges is an inhibitory role for TNX in a specific population of upper GI mechanosensory neurones, which may underlie fundamental aspects of sensory signal transduction at afferent endings. Our findings further indicate that these endings may be therapeutic targets for a range of symptoms in patients with TNX deficiency and may have a role in other patients such as those with hEDS.

## Methods

### Ethical approval

Full‐thickness gastric tissue (non‐pathological) was obtained from cancer patients (>10 cm away from tumors) undergoing gastrectomy using approved Human Research Ethics from Bart's and London NHS Trust with informed consent (NREC 09/H0704/2). Unfortunately, we could not obtain tissue from TNX deficient patients; however, TNX deficient patients gave theur written informed consent prior to completing questionnaires.

All studies were completed in accordance with the animal ethics policy and the study is in compliance the ethical principles required by *The Journal of Physiology* (Grundy, 2015). All mice used in these studies were killed by asphyxiation using carbon dioxide in accordance with the UK Home Office (Schedule 1, Animals Act 1986) for all experimental procedures. All human questionnaire studies were not registered in a database in accordance with the *Declaration of Helsinki*.

### Mouse tissue

Mice used in the present study originated from parent mice with a C57BL/6N background and was donated by Professor Manuel Koch (University of Cologne, Cologne, Germany). The generation of these mice has been described previously (Mao *et al*. 2002; Aktar *et al*. 2018). Knockout (KO) and wild‐type (WT) mice were phenotypically healthy, grew similarly and were of similar weight at the time of the study (WT: 19.9 ± 0.8 g; KO: 20.8 ± 1.1 g). All mice were reared and transported under conditions specified in the UK's Animal Welfare Act 2006. Mice aged between 10 and 12 weeks were used and were killed by a rising concentration of CO_2_ asphyxiation (Schedule 1, Animals Act 1986, U.K. Home Office).

### Immunohistochemistry

Immunolabelling with rabbit polyclonal TNX (dilution 1:200, sc‐25717, Santa Cruz Biotechnology, Santa Cruz, CA, USA), calretinin (dilution 1:500, CG1 and 6B3, Swant, Mountain View, CA, USA) and calcitonin gene‐related peptide CGRP (dilution 1:400, ab36001, Abcam, Cambridge, MA, USA; dilution 1:400; ABS026‐05‐02; Thermo Fisher, Waltham, MA, USA) was assessed in human and mouse specific stomach regions. Additionally, choline acetyl transferase (ChAT) (dilution 1:400, ab18736, Abcam) was used in brainstem sections.

#### Immunohistochemistry process

Tissue was fixed in 4% paraformaldehyde in 10 μm sections and then wholemounts were prepared and blocked in universal blocking serum (Dako, Glostrup, Denmark), followed by incubation overnight at 4°C with primary antibodies. Slides/whole mount tissue was then incubated for 1 h at room temperature with AlexaFluor conjugated secondary antibody (Invitrogen, Carlsbad, CA, USA) as appropriate. Slides/whole mounts were then mounted using a coverslip and Vectashield Hardset Mountant (Vector Laboratories, Inc., Burlingame, CA, USA) conjugated with 4′,6‐diamidino‐2‐phenylindole (H‐1500) and left to dry before viewing under the microscope at 40×.

### Vagal afferent recording studies

The methodology used was modified from Page *et al*. (2002) because whole stomach was used. Once a distension‐sensitive fibre was identified, 1 mL of Krebs solution was infused via a cannula into the mouse stomach for 1 min. After 1 min, the cannula was removed and the stomach was allowed to drain naturally. The empty stomach was then allowed to rest for 5 min before repeating this process two more times. Separate TNX‐KO mice were used for baclofen studies, where, after two distension recordings, baclofen (100 μm) was infused into the organ bath, as well as injected directly into the bath. After 10 min of baclofen infusion, two further distensions were made with a 5 min rest inbetween to measure the effects of baclofen on afferent firing. Gastric compliance was measured using a pressure transducer, which was attached to the cannula and used to infuse the stomach with standard volumes of Krebs solution. Single units were discriminated using wavemark analysis via Spike2 (Cambridge Electronic Design Ltd, Cambridge, UK).

### Gastric emptying studies

Mice were fasted overnight, placed in separate chambers, then consumed an egg yolk meal containing 1 μL 1 g^–1^ C^13^ octanoic acid (99% enrichment; Cambridge Isotope Laboratories, Andover, MA, USA). Once fully consumed, breath samples were collected at intervals up to 150 min and analysed for ^13^CO_2_/^12^CO_2_ using the isotope‐ratio mass spectrometer (Thermo Finnigan, Dreieich, Germany). Excretion data were analysed using non‐linear regression analysis for curve fitting to obtain gastric emptying half‐life (*t*_½_) and time taken for solid digestion into the duodenum (*T*_lag_) (Ghoos *et al*. 1993).

### Primary cell culture and calcium imaging of nodose ganglia

Primary cell cultures from the nodose ganglia of 12‐week‐old WT and TNX‐KO mice were prepared. Both nodose ganglia were removed under a stereomicroscope and placed immediately in cold F12 complete nutrient medium. The ganglia were dissociated by enzymatic digestion in two steps. First by collagenase II and dispase 3 mg mL^–1^ in Hank's balanced salt solution (HBSS) with agitation at 5 min intervals over 30 min and then by collagenase II alone for 15 min. After incubation, the ganglia were rinsed in cold HBSS and F12 and further dissociated by trituration using a fine pipette. Cells were then pelleted, rinsed in HBSS and resuspended in NeuroBasal A medium (Thermo Fisher). The cells were plated on poly‐d‐lysine coated 24‐well plates and placed in a 5% CO_2_ incubator at 37°C for 2 h to allow cell adherence. After 2 h, warmed complete NeuroBasal A medium (supplemented with B‐27, penicillin/streptomycin and glutamax) was added to each well and incubated for 48 h before the experiments. After 2 days of incubation, cell cultures were loaded with Fluo‐4‐AM to detect calcium flux 1 h before rinsing the cells with Krebs and reloading with Krebs. Changes in intracellular Ca^2+^ concentration [Ca^2+^] are reflected by the Fluo‐4‐AM fluorescence intensity and were recorded at 525/50 nm. Baseline activity of identified neurones was recorded for 120 s and then stimulated with either capsaicin (100 nm), cholecystokinin (CCK) (10 nm) or KCl (50 mm) for 120 s. Images were acquired every 5 s with a confocal LSM 880 with Airyscan using Zen software (Carl Zeiss, Oberkochen, Germany). Changes in flourescence intensity were measured by drawing a region of interest around neurones, and then measuring the change in fluorescence intensity. The total number of neurones that responded to stimuli was then averaged over a single time point. Peak intensity within the first 10 s was measured in cell cultures from both mouse groups. An identified neurone that changed in intensity as a result of the stimuli was described as a responder, whereas a non‐responder was described as neurone that showed no changes in fluorescence intensity. The total number of neurones that responded to capsaicin, CCK and KCl was noted individually. We sampled nodose ganglia neurones randomly to determine whether TNX deficient neurones respond differently as a population; more subtle changes may be revealed using patch clamp recordings from neurones retrogradely labelled from the stomach.

### Patient genotyping

Eleven patients (three males and eight females) identified from Radboud University Medical Centre took part and were genotyped for *TNXB* (Schalkwijk *et al*. 2001). Mutation analysis was performed using next‐generation sequencing testing *TNXB* DNA mutation, as described previously (Demirdas *et al*. 2016). Additionally, serum samples were analysed for TNX glycoprotein using an enzyme‐linked immunosorbent assay with rabbit anti‐TNX.

### GI symptom questionnaire

Patients with TNX deficiency completed a Flemish version of the validated Gastrointestinal Symptom Rating Scale (GSRS) (Svedlund *et al*. 1988). Questionnaire responses were translated and scored for five different domains: reflux, abdominal pain, constipation, indigestion and diarrhoea. Scores ranged from 1 (no symptoms) to 7 (unbearable symptoms). GSRS scores for abdominal pain and upper GI symptoms for reflux and indigestion were analysed in TNX deficient patients in comparison with a reference Swedish population of similar age and sex (Dimenas *et al*. 1996).

### Experimental design and statistical analysis

#### Immunohistochemistry

Qualitative images were obtained for both human and mouse stomach. Controls with no primary antibody were performed with each run of immunohistochemistry to confirm the specificity of the primary antibody, which resulted in no fluorescence above background.

A western blot with this antibody in humans revealed a band at the predicted weight of 268 kDa. Moreover TNX was absent in all TNX‐KO gut tissue. Images were obtained using Metamorph software (Molecular Devices, Sunnyvale, CA, USA) on an Olympus (Tokyo, Japan) MM Leica (Wetzlar, Germany) (sections) or Zen software on a LSM 710 or LSM 880 (Carl Zeiss) (confocal imaging wholemounts) at 40× magnification.

#### Vagal afferent recordings

In total, 12 WT and 12 KO mice (six females and six males in both groups) were used to measure spontaneous and post distension afferent activity in the stomach. Separate baclofen experiments in four WT *vs*. four KO mice (two females and two males in both groups) were also performed. Data were statistically analysed with an unpaired *t* test and individual data were plotted for all data sets with *P* < 0.05 being considered statistically significant.

#### Gastric emptying

In total, nine WT *vs*. 15 KO mice (five females and four males in WT and seven females and eight males in KO) were used. Statistical analysis was performed with an unpaired *t* test to compare between mouse groups and individual data were plotted with *P* < 0.05 being considered statistically significant.

#### Calcium imaging

In total, five WT *vs*. five KO mice (three females and two males in both groups) were used. In total, 25 WT and 17 KO individual nodose neurones were stimulated with KCL and capsaicin, respectively, whereas 18 WT and 34 KO individual nodose neurones were stimulated with CCK. Statistical analysis was performed using an unpaired *t* test. Individual data were plotted and *P* < 0.05 was considered statistically significant.

#### Patient questionnaires

In total, 11 patients (three males and eight females) with TNX deficiency were used and compared with a reference Swedish population (*n* = 2162). The reason for the small sample size in TNX deficient group is the small number of patients known to have TNX deficiency because it is uncommonly measured. Questionnaire data are shown as the mean ± SD. Statistical analysis was performed using an unpaired Student's *t *test for each symptom, with *P* < 0.05 being considered statistically significant.

All data are expressed as individual data points and variability within the data was represented using 95% confidence intervals of the mean. Statistical analysis was performed using Prism, version 7.02 (GraphPad Software Inc., San Diego, CA, USA).

## Results

### TNX localization in mouse and human stomach

**Figure 1:**
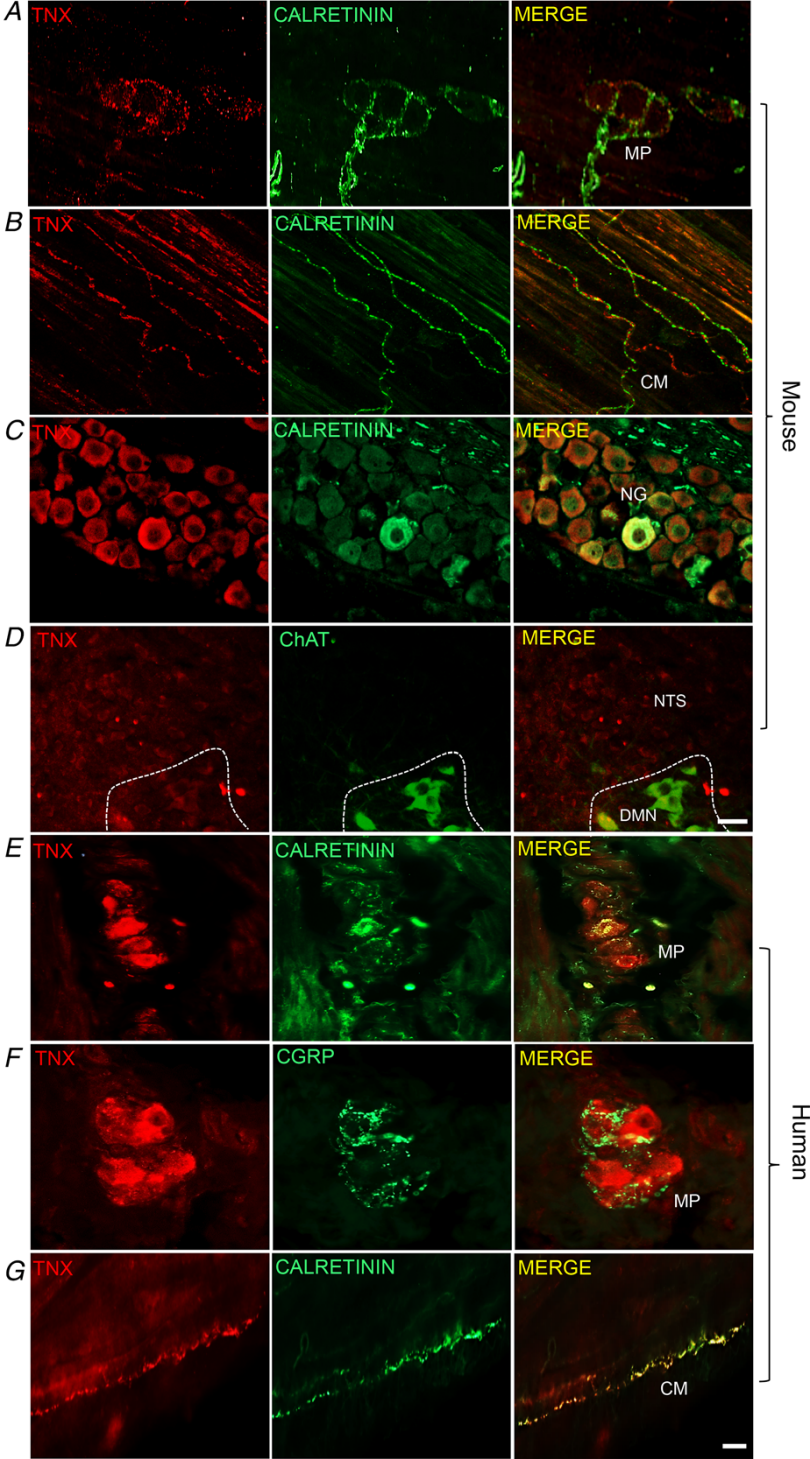
**TNX is expressed in neural structures in the stomach** Representative immunohistochemical images taken from whole mount mouse fundus labelled with TNX and calretinin in IGLE surrounding the MP but not in cell bodies (*A*). TNX‐immunoreactivity was commonly found in calretinin‐immunoreactive fibres in the smooth muscle layer of the stomach (*B*, merge). The cell bodies of vagal afferents in the nodose ganglia also showed positive TNX and calretinin co‐labelling in sections (*C*, merge). TNX positive endings found in the mouse nuclus tractus solitarius (NTS) (*D*). Unlike mouse, human fundus sections showed TNX‐ and calretinin‐immunoreactive cell bodies in the myenteric plexus (MP) (*E*, merge) that were distinct from CGRP fibres (*F*, merge). TNX also co‐labelled calretinin‐positive endings in the circular smooth muscle (*G*, merge). All images from mouse and human stomach are whole mount confocal z‐images except for nodose ganglia (NG) and circular muscle (CM) images, which were taken with an epifluorescence microscope. DMN, dorsal motor nucleus. Scale bars in (*A*–*D*) = 25 μm, (*E*–*G*) = 30.

Calretinin‐immunoreactivity is a well‐established marker for vagal afferents in rodent upper gut, and reliably labels IGLE and IMA (Berthoud *et al*. 1995; Fox *et al*. 2000; Phillips & Powley, 2000; Powley *et al*. 2013). In the mouse stomach, fibres positive for TNX+ calretinin were seen in circular muscle (Fig. 1 *B*). Fibres in stomach showed partially overlapping immunoreactivity for calretinin and TNX and corresponded anatomically and neurochemically to IMA (Fig. 1 *B*) and IGLE (Berthoud & Neuhuber, 2000). TNX and calretinin‐immunoreactive colocalization was also observed surrounding myenteric ganglia in IGLEs (Fig. 1 *A*), which have been previously described as tension‐sensitive vagal afferents (Berthoud & Powley, 1992; Phillips & Powley, 2000; Zagorodnyuk *et al*. 2001). Correspondingly, TNX‐immunoreactivity was found in a subpopulation of cell bodies of vagal afferent neurones in the nodose ganglia (Fig. 1 *C*) and more centrally in punctate endings within the nucleus tractus solitarius, with some in close proximity to dendrites of TNX‐negative ChAT‐positive neurones (presumably vagal preganglionic motorneurones) in the adjacent dorsal motor nucleus of the vagus (Fig. 1 *D*). Peripherally, qualitative analysis shows that some TNX‐immunoreactive cell bodies were present in gastric myenteric ganglia of human fundus (Fig. 1 *E*) but not in mouse (Fig. 1 *A*). CGRP did not co‐label with TNX in human fundus (Fig. 1 *F*). Similar to the mouse circular muscle, TNX‐immunoractive IMA were also found in human smooth muscle (Fig. 1 *G*).

### Gastric vagal afferent sensitivity in mice

**Figure 2:**
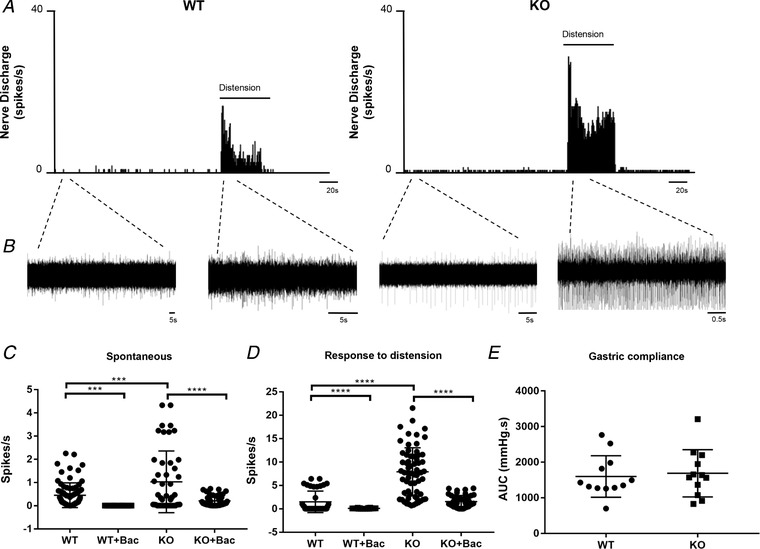
**Vagal afferent mechanosensitivity in WT and TNX‐KO mice** Responses to distension from single vagal afferent units innervating the stomach in WT *vs*. KO. Nerve discharge rate (histogram) was increased in KO in both spontaneous and distended (1 mL of fluid in stomach) conditions (*A*). *B*, corresponding raw trace to the nerve discharge rate. Spontaneous (*C*) (*P* = 0.0003) and distension (*P* < 0.0001) induced afferent firing (*D*) was significantly increased in TNX‐KO compared to WT, (*n* = 14 WT afferent units *vs. n* = 18 KO afferent units). Addition of baclofen (100 μm) significantly reduced both spontaneous (*P* < 0.0001) and distension (*P* < 0.0001) induced firing back to levels similar to those observed in WT mice (*C* and *D*) (*n* = 23 KO afferent units). Baclofen also reduced WT spontaneous (*P* < 0.0001) and distension (*P* = 0.028) induced firing (*C* and *D*) (*n* = 22 WT afferent units). There was no change in gastric compliance to 1 mL of Krebs over 1 min in WT *vs*. TNX‐KO mice (*n* = 12 WT *vs. n* = 12 KO). *E*, statistical analysis was performed using an unpaired *t* test.

The pattern of TNX localization in calretinin‐immunoreactive endings in the stomach suggested a role in vagal afferent (IGLE and IMA) function; therefore, we investigated whether there was an alteration in mechanosensory and electrophysiological properties of gastric vagal afferents that lack TNX. Single afferent fibre recordings of gastric tension receptors that are mechanosensitive to contraction and distension exhibiting slow adaptation to innocuous wall tension (Page *et al*. 2002) were analysed. Spontaneous firing was significantly increased by 66% in TNX‐KO compared to WT (0.23 ± 0.06 WT *vs*. 0.69 ± 0.08 KO, *P = *0.0003) (Fig. 2 *C*). Similarly, responses to distension were significantly increased in TNX‐KO (2.37 ± 0.67 WT *vs*. 9.67 ± 1.2 KO, *P < *0.0001) (Fig. 2 *D*). Distension‐induced changes in intragastric pressure showed no difference in the area under curve (1598.9 ± 168.2 WT *vs*. 2055.6 ± 177.9 KO, *P = *0.7326) (Fig. 2 *E*). Therefore, gastric compliance is unaltered, suggesting increased afferent sensitivity in TNX‐KO is a property of the afferent neurones and not their environment.

### Rescue of afferent hypersensitivity in TNX‐KO mice

Addition of the GABA_B_ receptor agonist baclofen caused a significant reduction in spontaneous firing (0.69 ± 0.08 KO *vs*. 0.13 ± 0.029 KO + baclofen, *P < *0.0001) (Fig. 2 *C*) and afferent firing during distension in TNX‐KO (9.67 ± 1.2 KO *vs*. 1.55 ± 0.253 KO + baclofen, *P < *0.0001) (Fig. 2 *D*). Afferent firing post baclofen was reduced in TNX‐KO mice similar to levels observed in WT mice (Fig. 2 *C*).

### Gastric emptying

**Figure 3:**
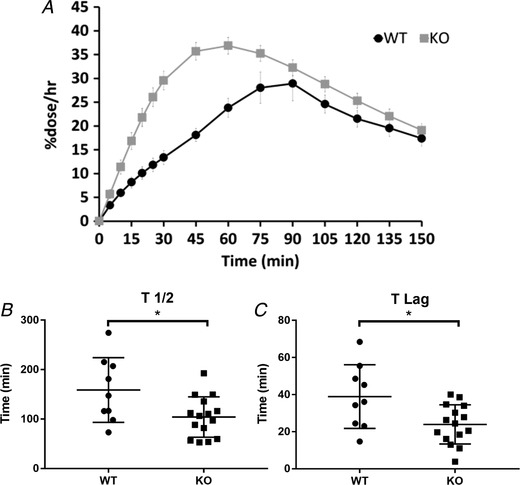
**Gastric emptying in WT and TNX‐KO mice** ^13^CO_2_ excretion curve in WT *vs*. KO. KO curve (grey) showing an increase in gastric emptying rate because the curve has shifted to the left (*A*). The half‐life (*t*_½_) was significantly reduced in the KO (103 ± 12.9 min; *P* = 0.0277). *B*, time taken for solid food to be broken down (*t*_lag_) was significantly reduced in the KO (24 min ± 2.7; *P* = 0.0194) (*C*) (*n* = 9 WT *vs. n* = 15 KO). Statistical analysis was performed using an unpaired *t* test.

Because vagal afferents showed mechanical hypersensitivity in the absence of TNX, we investigated whether translated to reflex control of gastric motility by observing gastric emptying in TNX‐KO. In WT, a gradual increase in labelled CO_2_ ultimately peaked at 90 min, whereas TNX‐KO mice had a rapid increase that peaked at 60 min (Fig. 3 *A*). Starting at 100 min, CO_2_ gradually declined in WT mice, unlike TNX‐KO that had a rapid decline at 75 min, demonstrating faster gastric emptying (Fig. 3 *A*). The *t*_½_ (158 min ± 21.8 WT *vs*. 103 min ± 12.9 KO, *P = *0.0277) (Fig. 3 *B*) and *t*_lag_ (24 min ± 2.7 WT *vs*. 38 min ± 5.7 KO, *P = *0.0194) (Fig. 3 *C*) were significantly reduced in TNX‐KO, suggesting that propulsive motility of the stomach is enhanced.

### Excitability of cultured nodose ganglia

**Figure 4:**
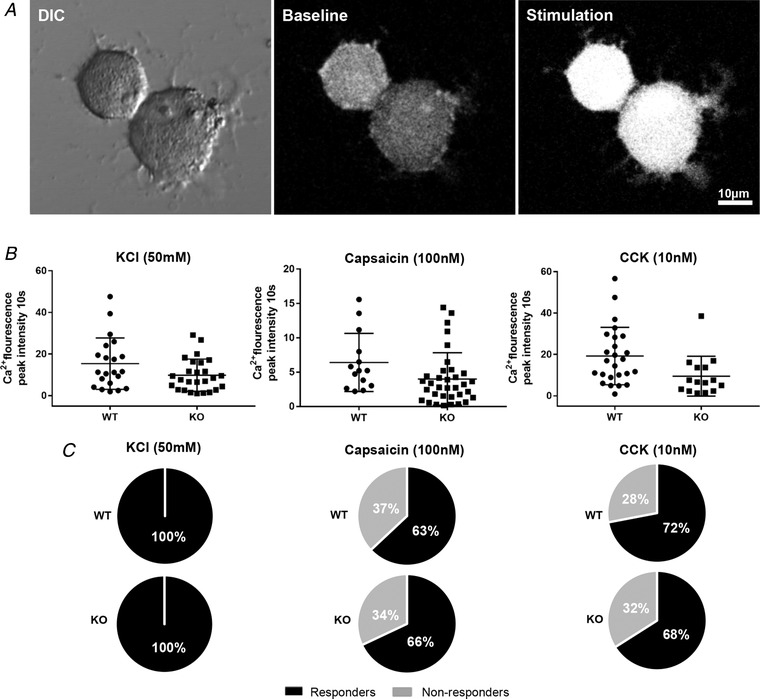
**Capsaicin and CCK effects in cultured nodose neurones** Differential interference contrast image of neurones in culture, at baseline and stimulated neurone (*A*). The average peak intensity within the first 10 s shows no change in response to KCl, capsaicin or CCK (*B*). Numbers of responsive and non‐responsive neurones show no change in WT and KO with KCl, capsaicin or CCK stimulation (*C*) (*n* = 25 WT *vs. n* = 17 KO for KCL/capsaicin experiments and *n* = 18 WT *vs. n* = 34 KO for CCK experiments).

Vagal afferent hypersensitivity could be the result of an altered generation of action potentials by mechanical stimuli at the endings or by altered excitability of the neurone as a whole. To determine which is most probable, nodose ganglia neurones were isolated from their target and studied in culture. They grew similarly (Fig. 4 *A*) in both WT and KO mice, indicating a lack of influence of TNX on cytoarchitecture and viability. Responsiveness was assessed by peak calcium responses within the first 10 s post‐KCl, CCK and capsaicin, which showed no differences between WT or TNX‐KO neurones (Fig. 4 *B*). Additionally, there were no differences in the number of responders *vs*. non‐responders to KCl, CCK and capsaicin between groups (Fig. 4 *C*). Thus, the changes that we observed are most probably a result of mechanical hypersensitivity.

### Gastrointestinal symptoms in TNX deficient patients

**Figure 5:**
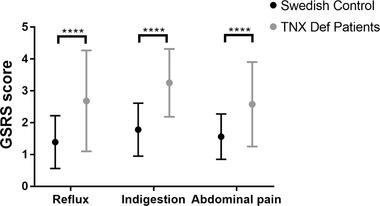
**Gastrointestinal symptoms in TNX deficient patients and healthy controls** The severity of gastrointestinal symptoms assessed using the GSRS scores (means and 95% confidence interval) in patients with TNX deficiency (*n* = 11) and healthy controls (*n* = 2162). Abdominal pain, reflux and indigestion were significantly higher in TNX deficient patients compared to Swedish controls (^****^ *P* < 0.0001). Statistical analysis was performed using a two‐way ANOVA (Sidak multiple comparison test).

TNX deficient patients showed significantly increased severity of GI symptoms overall (*P *< 0.0001) compared to healthy controls (Fig. 5). Specifically, symptoms of gastroesophageal reflux, abdominal pain and indigestion were all increased significantly.

## Discussion

Having recently discovered a novel role for TNX in enteric neurones of the lower GI tract, it followed that there was probably also a role in the upper gut, particularly in view of the fact that patients with TNX deficiency have both upper and lower GI symptoms. We found a strong correlation between TNX gene deficiency and upper GI symptom severity in a patient cohort. Correspondingly, TNX was normally present in calretinin‐immunoreactive structures, analogous to vagal afferent endings in both humans and mice. Electrophysiological recordings showed that TNX KO mice have greatly enhanced sensitivity of vagal afferents to gastric distension. This is associated with accelerated gastric emptying *in vivo*, and matches the upper GI symptoms reported by patients. We also show that increased afferent function can be reversed by activation of inhibitory GABA_B_ receptors on gastric vagal afferents. Thus, TNX plays an inhibitory role in a specific population of upper GI mechanosensory neurones, and may underlie aspects of sensory signal transduction at afferent endings. Our findings further indicate these endings may be therapeutic targets for a range of symptoms in patients with TNX deficiency and may have a role in other patients such as those with hEDS.

**Figure 6:**
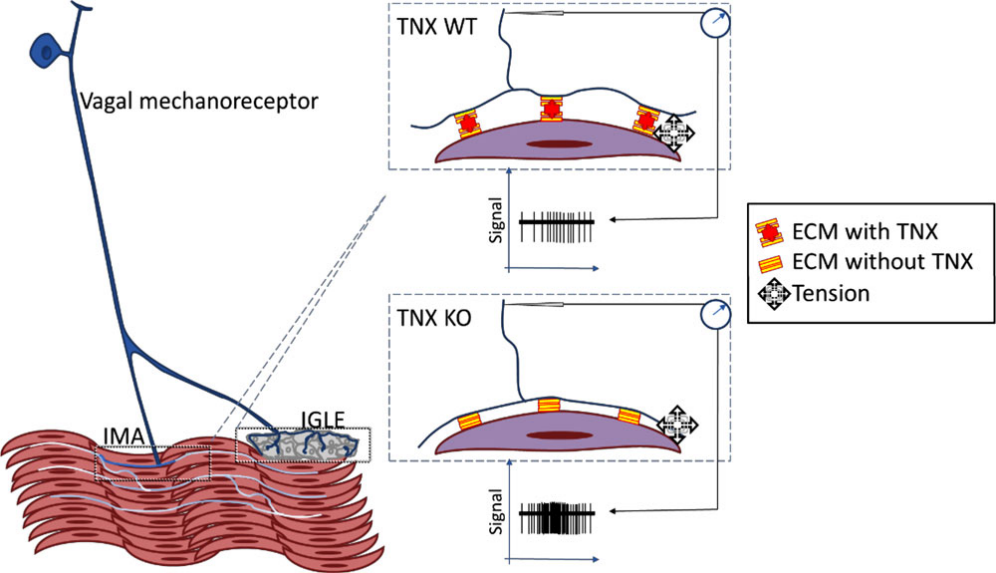
**Hypothesized anti‐adhesive mechanisms by which TNX supports sensory function** In the stomach, vagal afferent endings are maintained at an optimal attitude to the host tissue by TNX, whether that be ganglia or muscle, or possibly interstitial cells (not shown). Without TNX, the ECM is improperly supported and so excessive strain, both resting and particularly active, is exerted on the mechanosensitive elements of the afferent ending.

TNX‐immunoreactivity was found in a small population of nodose ganglion neurones and in sparse central vagal terminals in the nuclus tractus solitarius. Therefore, we corroborated our finding in hindgut indicating that TNX is expressed by GI neurones, although with the exception that expression in upper gut occurs in extrinsic, rather than intrinsic neurones. This indicates a very different role for TNX in the upper GI tract. TNX‐immunoreactivity was seen often in endings that colocalized with calretinin. It is established from other work that these are vagal afferent terminals with cell bodies in the nodose ganglia (Berthoud *et al*. 1995; Fox *et al*. 2000; Phillips & Powley, 2000; Powley *et al*. 2013), which fits with the lack of TNX‐immunoreactive intrinsic cell bodies. Correspondingly, based on this finding of TNX expression in extrinsic neurones, it was important to determine whether TNX influences vagal afferent function, which we investigated with a gastric vagal afferent preparation adapted from those used prevously (Page *et al*. 2002) to reveal differences between genotypes. We observed greater mechanosensitivity and baseline discharge of individual low‐threshold distension‐sensitive afferents, known as tension receptors (Page *et al*. 2002). This was in the absence of changes in gastric compliance, suggesting that it was a property of the nerve endings only. This notion was further supported by studies of nodose ganglion cell bodies, which showed no difference in responsiveness after application of KCl, CCK or capsaicin. This leads us to assume that TNX plays a specialized role in the anchoring of nerve terminals to their targets, perhaps by allowing flexibility between the two. Thus, without TNX, the afferent ending becomes less dynamic in its attachment and reaches full mechanical distortion with less movement relative to the surrounding tissue. As shown in Fig. 6, the spacing of mechanosensory terminals and smooth muscle elements is critical for normal afferent signalling. It will require further studies with electron microscopy to determine whether this anchoring role is tenable. In our previous study of high threshold nociceptive colonic afferents in the splanchnic nerves (Aktar *et al*. 2018), we also found increased mechanosensitivity in the TNX‐KO, although TNX labelling was not associated with the endings themselves and was mutually exclusive with their marker: CGRP. In that case, there was ∼50% greater mechanosensitivity in the KO, whereas, in the present study, we found a 400% greater mechanosensitivity, which is perhaps not unexpected given the direct association of TNX with vagal afferents compared to the distinct location of TNX relative to splanchnic afferents. This is by far the biggest difference in vagal function that we have seen in several comparisons of KOs of sensory genes with WTs, suggesting that the role of TNX is critical. The indirect role of TNX in splanchnic afferents is supported by the fact that they appeared to sprout into the otherwise TNX‐negative mucosa (Aktar *et al*. 2018) (suggesting a barrier function), whereas their direct role in vagal afferents is supported by the observation that their anatomical relation to other tissues was not noticeably altered in TNX‐KO, although their function was markedly different.

The main expected consequence *in vivo* of increased gastric vagal afferent sensitivity, upon reaching the central nervous system (CNS) would be increased perception of non‐nociceptive mechanical forces in the stomach, although mouse models are not available for this. Therefore, we investigated another major consequence of vagal afferent activation, which is the control of gastric emptying (Browning *et al*. 2014). This is mediated via a vago‐vagal reflex in the brainstem, where we saw localization of TNX presumably at the central endings of vagal afferents. It is known that distension of the proximal stomach promotes motor activity of the distal stomach via this reflex, which serves to augment the antral pump, in turn facilitating the emptying of solids from the stomach through the pylorus into the duodenum (Grundy *et al*. 1989), (Andrews *et al*. 1980). There was a faster rate of gastric emptying of a solid meal in TNX‐KO that was comparable to the greater afferent sensitivity, which supports a role for TNX in this pathway *in vivo*. Although studies of gastric emptying specifically in TNX‐deficient patients are lacking, some studies of emptying in ungenotyped hEDS patients show increased solid emptying (Menys *et al*. 2017), suggesting the same phenomenon. By comparison of GSRS questionnaire data, we were able to determine whether TNX‐deficient patients reported upper GI symptoms differently from to controls, and we found that, in cases of the perception of indigestion and reflux, scores were approximately double in the TNX‐deficient cohort compared to controls. This is in addition to the lower GI symptoms that we already reported in this cohort.

The data would suggest that all upper GI symptoms in TNX‐deficient patients (and possibly therefore those reported in a large number of hEDS patients) may have a common origin in the hypersensitivity of vagal afferents. In that case, it would make these endings a potential therapeutic target. This has been suggested previously, when the triggering of transient lower oesophageal sphincter relaxations by vagal afferents via a similar pathway was exploited as a therapeutic target for gastro‐oesophageal reflux disease (Tonini *et al*. 2004). Specifically, the expression of GABA_B_ receptors by vagal afferents was exploited as a means of reducing their excitability, and thus the strength of their signal to the central motor programme giving rise to sphincter opening that allows acid reflux (Zhang *et al*. 2002). This was carried out almost successfully in clinical trials, despite the lack of any evidence for hypersensitivity of vagal afferents in gastro‐oesophageal reflux disease. In the present study, however, we have a demonstrable hypersensitivity that is specific to mechanical stimuli, which we know is susceptible to inhibition via GABA_B_ receptors. Therefore, we aimed to rescue the hypersensitivity of vagal afferents using the prototypic GABA_B_ receptor agonist baclofen. This was highly effective, with profound inhibition of responses to distension, as well as spontaneous activity. Unfortunately, baclofen has considerable effects on the CNS (Dario *et al*. 2007) and so it was not possible to determine whether these effects seen in the organ bath were translatable to *in vivo* experiments on gastric emptying. For that, a more peripherally restricted compound would be required.

Generally speaking, mouse and human data were comparable in the present study in terms of differences between TNX‐deficient and control cohorts. However, not all parameters could be assessed, such as gastric emptying in TNX‐deficient patients (for ethical reasons related to their primary referral) and upper GI symptoms in mice (because of lack of outcome markers). In the case of TNX localization, we were able to compare directly the two species and we did find discrepancy. The mouse lacked completely any somatic labelling in gastric myenteric neurones for TNX, whereas human specimens invariably showed that gastric myenteric neurones were TNX‐immunoreactive, albeit at a much lower abundance than those in the lower GI tract. This may suggest that the neuronal subtypes may differ between species and, indeed, it has been noted previously that very few or no after‐hyperpolarization (AH)/Type II myenteric neurones are found in the upper GI tract of some species but not others (Mazzuoli & Schemann, 2012). Of importance is the observation that larger mammals more probably possess these neurones, which means that TNX‐immunoreactivity may be serving as a marker for this population, along with calretinin (although not exclusively). This would fit with the far higher prevalence of TNX + calretinin‐immunoreactive enteric neurones in the colon, where AH/Type II neurones are common in all species. It was not possible to observe fine anatomical detail of the neurones labelled in the present study, and therefore their Dogiel type, although the colocalization of TNX with calretinin is a further indicator that this is their phenotype. Intrinsic AH/Type II neurones more closely resemble extrinsic sensory neurones than other functional subtypes in the enteric nervous system, and so it may be that TNX associates exclusively with a non‐nociceptive sensory phenotype because vagal afferents are also sensory neurones.

Perineuronal nets (PNN) are a feature of central synapses, and are formed by the interaction of another tenascin, tenascin‐R, with various extracellular matrix (ECM) components (Sorg *et al*. 2016). PNN are involved in structural connections between neurones and in synaptic plasticity (Kwok *et al*. 2011). We have no indications from our data as to whether or not TNX fulfils such a role in the gut or in the dorsal vagal complex because labelling of other ECM components is required to do this definitively. However, it is intriguing how IGLE form a basket‐like network around myenteric ganglia, which may serve a structural role in the delineation of discrete ganglia in the enteric nervous system. The location and orientation of IGLE and IMA would simultaneously assist in their mechanosensory function by detecting force impinging between and within smooth muscle layers.

Could differences in afferent endings lacking TNX reflect a role for TNX in correct spacing of afferent terminals relative to host tissue? By comparison, the ECM molecule agrin is considered to be important in forming neuromuscular junctions and maintaining synapses between cholinergic preganglionic axons and sympathetic neurons (Gingras *et al*. 2002). Mice lacking a part of the agrin molecule show marked reduction in acetylcholine receptor, which indicates that this molecule is crucial in normal synapse function in the CNS (Gingras *et al*. 2002), (Gautam *et al*. 1996). Moreover, agrin increases nicotinic transmission at the synapse by modulating the space between the gap junction‐mediated electrical coupling (Martin *et al*. 2005). Similar to agrins, TNX may have a role in modulating the vagal afferent microenvironment by regulating the intercellular spaces between the vagal afferent terminals and smooth muscle or interstitial cells of Cajal. Therefore, TNX probably plays a similar role to other ECM proteins with respect to modulating neural connectivity. Perhaps TNX is important in maintaining the shape of vagal afferent endings; therefore, the absence of TNX may transduce mechanical forces less dynamically. The morphology of vagal afferent endings in the KO model was not altered and therefore this needs to be explored further at the ultrastructural level.

In TNX deficiency, in addition to extracellular abnormalities, ion channels that detect mechanical sensitivity may be altered, thus affecting the movement of sodium influx initiating the action potential. This change could then result in increased afferent firing observed in the KO. Interestingly, TNR is shown to modulate the activity of sodium channels that are involved in action potential generation (Weber *et al*. 1999), in particular cells containing β1 and β2 subunits (Xiao *et al*. 1999). TNR deficient mice show no change in the distribution of sodium channels, although action potentials recorded from optic nerves showed a significant decrease in conduction velocity (Weber *et al*. 1999). Because TNX and TNR are within the same family, the functional importance of voltage‐gated sodium channels may be affected similarly by their removal.

In conclusion, we propose that TNX serves at least two important roles in gastrointestinal neural function, in addition to its established role in the structure and rigidity of somatic tissues. These are: (i) a positive influence on cholinergic neurotransmission in intrinsic enteric neurones in the intestine and (ii) a negative influence on mechanical coupling of vagal afferent endings in the stomach. These apparently opposite effects may both ensue from changes in ultrastructure.

## Additional information

### Competing interests

The authors declare that they have no competing interests.

### Author contributions

All authors had access to the study data and reviewed and approved the final manuscript. Professor Aziz and Professor Blackshaw are joint senior authors. LAB, QA and RA were responsible for the study concept and design. RA, MP, EJA, CB and NCV were responsible for acquisition of data. RA, LAB, MP and SE were responsible for analysis and interpretation of data. RA was responsible for drafting the manuscript. LAB, QA and RA were responsible for critical revision of the manuscript for important intellectual content. RA and MP were responsible for the statistical analysis. LAB and QA obtained funding. AF, AM, SE, SK, SK, CB, NCV and MP were responsible for administrative, technical or material support. LAB, QA, MP and NCV were responsible for study supervision. All authors approved the final version of the manuscript submitted for publication, and all persons designated as authors qualify for authorship and all those who qualify for authorship are listed.

### Funding

This work was funded by a Wellcome Trust University Award and a Henry Smith Charity Grant to Professor L. A. Blackshaw, a Bowel and Cancer Research PhD studentship to Dr R. Aktar, and an Ehlers Danlos Society Award to Professor Aziz.
